# Optimizing therapeutic drug monitoring of mirtazapine — applying therapeutic reference range, concentration–dose ratio/dose-related concentration, and metabolic ratio in a naturalistic setting

**DOI:** 10.1007/s00228-025-03907-6

**Published:** 2025-08-20

**Authors:** Kajetan Nierychlewski, Michael Paal, Sebastian Meinzer, Richard Musil, Markus Schwarz

**Affiliations:** 1 Institute of Laboratory Medicine, LMU University Hospital, LMU Munich, Munich, Germany; 2 Department of Psychiatry and Psychotherapy, LMU University Hospital, LMU Munich, Munich, Germany; 3 Oberberg Fachklinik Bad Tölz, Bad Tölz, Germany

**Keywords:** Mirtazapine (MIR), Therapeutic drug monitoring (TDM), Therapeutic reference range (TRR), Dose–related concentration (DRC), Metabolic ratio (MR), Concentration–dose ratio (C/D)

## Abstract

**Purpose:**

Mirtazapine is a common antidepressant, regularly monitored through therapeutic drug monitoring. The previously published Consensus Guidelines in Neuropsychopharmacology provide data on the therapeutic reference ranges (TRR), dose-related concentrations (DRC), and metabolic ratios (MR). We aimed to investigate these TDM ranges in a real-world naturalistic setting to offer more practical, evidence-based recommendations.

**Methods:**

In a retrospective single-center cohort study, we screened 361 patients with major depressive disorder undergoing mirtazapine treatment. Following the Consensus Guidelines (Hiemke et al., 2017), we compared three TDM tools in combination: (I) the TRR, (II) the concentration-to-dose (C/D) ratio with DRC factors, and (III) the MR.

**Results:**

We analyzed 328 patients (mean age 54 ± 16 years; 42% female). Serum levels of mirtazapine were below the TRR in 38% and above in 8%. Women exhibited an 18% higher C/D ratio compared to men, and elderly patients (> 65 years) demonstrated a 27% higher median C/D ratio than younger individuals. DRC values differed from the guideline-recommended ranges, likely due to higher dosing, unexpectedly lower serum values, interindividual pharmacokinetic variability, genetic polymorphisms, or polypharmacy.

The median MR was 25% higher in women compared to men. Notably, 13 samples (4%) went beyond the designated MR range (either < 0.2 ng/mL or > 1.2 ng/mL).

**Conclusions:**

This study provides real-world insights into established DRC values of mirtazapine. Observed age- and sex-related pharmacokinetic differences highlight the need for individualized dosing strategies, while further multicenter studies are needed to confirm their reproducibility.

## Background

Depression is a frequent and growing health condition, with a reported prevalence of 6% in Europe in 2021 [1]. Due to necessary prolonged or repeated hospitalizations, it imposes a significant burden on healthcare systems worldwide [2]. Treatment involves pharmacotherapy with antidepressants in addition to psychotherapy and sociotherapeutic interventions [3, 4]. Mirtazapine (MIR) is a second-generation tetracyclic antidepressant commonly administered to treat major depression due to its advantageous therapeutic and side-effect profile [5]. MIR works as a noradrenergic and specific serotonergic antidepressant [6]. Its unique pharmacological profile involves creating antagonistic effects on a2–adrenergic auto– and heteroreceptors, 5–HT2 and 5–HT3 serotonin receptors, as well as antagonism at histamine H1 receptors [7–9]. MIR undergoes two main metabolic transformation pathways. The primary route involves cytochrome P450 (CYP) 3A4–mediated conversion to N–desmethylmirtazapine (NDM), while the secondary pathway is facilitated by CYP2D6 and CYP1A2, leading to 8–hydroxymirtazapine (8–OH–MIR) [10, 11]. The ultimate ratio of NDM to 8–OH–MIR is approximately 32:1 [12]. NDM serves as the primary pharmacologically active metabolite, with activity levels approximately 3 to 4 times lower than those of the parent compound [7].

In clinical practice, therapeutic drug monitoring (TDM) is crucial, particularly in cases involving notable pharmacokinetic variability or a narrow therapeutic range [13]. TDM’s advantages have been demonstrated when treatment fails to provide substantial relief from symptoms [14] or causes severe side effects during the initial dosing stage, such as constipation, weight gain, dry mouth, drowsiness, excessive sedation, and fatigue [15, 16]. The primary objective of TDM is to enhance and personalize pharmacological treatments by quickly identifying appropriate dosage levels, promoting better treatment adherence, while minimizing side effects [14, 17–19]. For MIR, TDM is especially beneficial due to its CYP3A4–mediated metabolism, which is susceptible to drug–drug interactions caused by enzyme induction or inhibition, while being only minimally affected by genetic variability [16, 20].

Based on the published guidelines, it is recommended to assess a certain set of TDM tools, including the (I) therapeutic reference range (TRR), (II) concentration–to–dose (C/D) ratio, including the dose–related concentration (DRC) factors, and (III) metabolic ratio (MR).

(I) TRR can help clinicians adjust the dosage to maintain therapeutic efficacy while minimizing potential side effects related to concentration changes. It defines a concentration range in which the intended drug effect is achieved [21, 22]. Consequently, concentrations below the lower threshold indicate a diminished therapeutic response, and concentrations above the upper threshold imply no further improvement in effectiveness. However, maintaining drug levels within this range can be challenging due to pharmacokinetic variability, particularly the influence of metabolic pathways. CYP3A4 inducers, such as carbamazepine, can increase the clearance of MIR, potentially up to twofold [23]. In contrast, CYP3A4 inhibitors, such as ketoconazole, may reduce the clearance of MIR, potentially leading to increased plasma levels [20]. Ketoconazole has been shown to significantly inhibit CYP3A4–mediated metabolism of MIR, supporting its role as a relevant interaction partner [24]. These significant pharmacokinetic interactions suggest that TDM could be valuable in optimizing MIR therapy, especially when co–administering drugs that affect CYP3A4 activity.

(IIa) The C/D ratio assesses pharmacokinetic variability and considers individual dosing [10, 25, 26]. Based on the study by Tveit et al. [27], MIR shows significant age– and sex–related differences in C/D ratios. The C/D ratios for MIR increase at age 44–55 years, with a doubling estimated to occur at 90 years of age [27]. Additionally, women exhibit higher C/D ratios compared to men, indicating that females may require lower MIR doses to achieve similar serum concentrations [27]. Further cases, such as the interaction between voriconazole and immunosuppressants [28, 29] or differences in clozapine metabolism across populations [30], illustrate its significant impact in investigating pharmacokinetic abnormalities and identifying potential drug–drug interactions [30, 31].

(IIb) Improvements in TDM enabled the integration of patient-specific dosages based on pharmacokinetic principles, and as part of this progress, the concept of DRC was evaluated to provide an initial estimate of the relationship between drug dosage and serum concentration [19, 32, 33]. Hiemke et al. [19] presented a comprehensive yet practical approach for estimating expected changes in drug concentrations, aiming to bridge the gap between pharmacological knowledge and its application in clinical practice. The DRC factors enable the comparison between a measured drug concentration and a theoretically projected range of drug concentrations. The average steady-state concentration of a drug can be computed when the daily maintenance dose, dose interval, total clearance, and bioavailability are known. Multiplying the DRC factors by the daily dose can provide insights into abnormal metabolic patterns.

(III) A further implementation was the MR, defined as the ratio of metabolite concentration to parent compound concentration, which potentially reflects the enzyme’s activity [19, 24, 26]. Current research often disregards the associations with metabolite concentrations, even though outliers from the anticipated MR ranges may point towards partial non-adherence or abnormalities in drug metabolism, requiring further clarification [34, 35].

Previous studies have already explored the pharmacokinetics of MIR in naturalistic settings, examining variables such as age, weight, sex, drug interactions, smoking behavior, and CYP genotype, but they face limitations in the use and integration of mentioned TDM tools [10, 25–27, 36–38]. This study investigated the combined use of the advanced TDM tools TRR, C/D ratio, DRC, and MR in a naturalistic cohort of patients treated with MIR for major depression. Applying these tools aimed to provide recommendations on realistic ranges of values and validate their practical utility in optimizing personalized pharmacotherapy to strengthen collaboration between clinicians and pharmacologists for future clinical practice.

## Methods

### Study subjects and design

This study was conducted as a single-center, retrospective cohort study at a tertiary academic center (LMU University Hospital, Munich, Germany). It was approved by the local ethics committee (LMU Munich, project number 365–15, 12.08.2015) and performed in accordance with the Declaration of Helsinki. We included inpatients who were treated at the psychiatric clinic from 2013 to 2016 and underwent routine laboratory measurements for evaluation of MIR and NDM concentration, with drug administration taking place under supervision. Blood sampling was performed at steady-state trough levels to ensure reliable TDM assessment. Blood samples were routinely collected in the early morning, prior to the first daily dose and following at least 1 week of continuous dosing to ensure steady-state trough levels, using S–Monovette® neutral Z, 7.5 mL serum tubes without gel separators (Sarstedt, Germany). Inclusion criteria were an age of 18 years or older, the diagnosis of a depressive disorder according to the ICD–10 of the Classification of Mental and Behavioral Disorders (F31.3–5, F32, F33, F34, F38) [39], MIR intake of 15 to 45 mg daily, and available MIR and NDM measurement. For patients with multiple MIR serum concentrations, only the first reported value was included per patient. In total, 33 patients were excluded due to deviating MIR dosage (below 15 mg or exceeding 45 mg), missing TDM data, and one patient had MIR and NDM concentrations below the lower limit of quantification. Data on polypharmacy were not systematically collected, and medication dosages were not considered in detail. Instead, only patients receiving medications known to have significant interactions with MIR, such as carbamazepine, ciprofloxacin, fluoxetine, bupropion, and haloperidol, were included in the main analysis, ensuring a targeted evaluation of polypharmacy’s impact on MIR pharmacokinetics. These medications were selected due to their established effects on CYP3A4 or CYP2D6, which are involved in MIR metabolism [16]. To assess MIR pharmacokinetics without the impact of CYP–interacting co-medications, a subgroup analysis was performed, excluding 56 patients (17% of the total cohort) who were receiving drugs with known CYP3A4 or CYP2D6 interaction potential. Smoking status was documented for all patients and included in the clinical dataset. Additional analyses were performed to explore potential associations between smoking and MIR pharmacokinetic parameters, specifically the C/D ratio and DRC.

#### Analytical method

MIR and its metabolite NDM were quantified using a Liquid Chromatography–Tandem Mass Spectrometry (LC–MS/MS) system, recognized as the gold standard for TDM. Analysis of the samples was conducted on an Acquity ultra–HPLC system with a triple quadrupole MS Xevo TQ–S (Waters, Milford, MA, USA). The chromatographic separation was performed using a PerfectSil Target ODS–3 HD column (100 mm × 2.1 mm) with a particle size of 5 μm and with a total run time of 7 min. Mobile phase A consisted of water and formic acid (99.9:0.1, v/v) plus 10 mM ammonium formate and phase B of acetonitrile, both running at a flow rate of 0.75 ml/min in step dilution mode [40]. The method demonstrated high reliability, with consistently low intra- and interday inaccuracy and imprecision. In detail, intra- and inter-assay imprecision remained below 3% for both MIR and NDM across low, medium, and high quality control levels. The lower and upper limits of quantification were 4.5 ng/mL and 409.5 ng/mL for MIR, and 4.4 ng/mL and 399.6 ng/mL for NDM, respectively. Calibration was linear over the validated range, with correlation coefficients (*r*) of 0.999 for MIR and 0.9991 for NDM, indicating excellent linearity (MIR: y = 1.001x – 0.101; NDM: y = 0.998x + 0.182).

### Statistical analysis

Statistical analyses and graph generation were performed using IBM Statistical Package for the Social Sciences (SPSS, version 29.0). Continuous variables with non-normal distributions were reported as medians. Spearman’s rank correlation test was employed to assess associations between the C/D ratio, MR with continuous variables. The non-parametric Mann–Whitney *U* test was used for all group comparisons, including those involving gender and smoking status due to non-normal data distribution. A chi-square test was used to examine the association between daily MIR dose and serum MIR concentrations according to TRR status. A *p*-value < 0.05 was considered statistically significant. Analyses were conducted in a naturalistic, hypothesis-generating context without a predefined primary hypothesis. Variables included in the analysis were selected based on prior literature. Only univariate tests were performed, and given the limited number of comparisons, no correction for multiple testing was applied.

## Results

Baseline characteristics are presented in Table 1. Mean age was 54 years (SD 16 years) and 42% (139/328) were female.

**Table 1:** Patient characteristics

Parameter	Total (*n* = 328)
Age (years), mean (SD)	54 (16)
Age < 65 years, *n* (%)	239 (73)
Age range (years)	18–90
Female, *n* (%)	139 (42)
Smoker, *n* (%)	107 (33)
Mirtazapine	
Dose (mg), mean (SD)	32.9 (10.5)
Dose (mg), median (IQR)	30.0 (30.0–45.0)
Dose range (mg)	15.0–45.0
conc. MIR (ng/mL), mean (SD)	42.2 (26.9)
conc. MIR (ng/mL), median (IQR)	36.8 (25.0–52.5)
conc. MIR (ng/mL), range	4.5–153.0
conc. NDM (ng/mL), mean (SD)	20.6 (12.6)
conc. NDM (ng/mL), median (IQR)	17.7 (11.5–25.8)
conc. NDM (ng/mL), range	4.5–84.9

*IQR* interquartile, *MIR* mirtazapine, *n* number of patients, *NDM* N–Desmethylmirtazapine, *SD* standard deviation

We evaluated the MIR serum concentrations of the cohort with the TDM tools (I) TRR, (II) C/D ratio and DRC, and (III) MR. In our cohort, patients demonstrated a mean daily MIR dose of 32.9 mg.

### (I) TRR

**Fig. 1:**
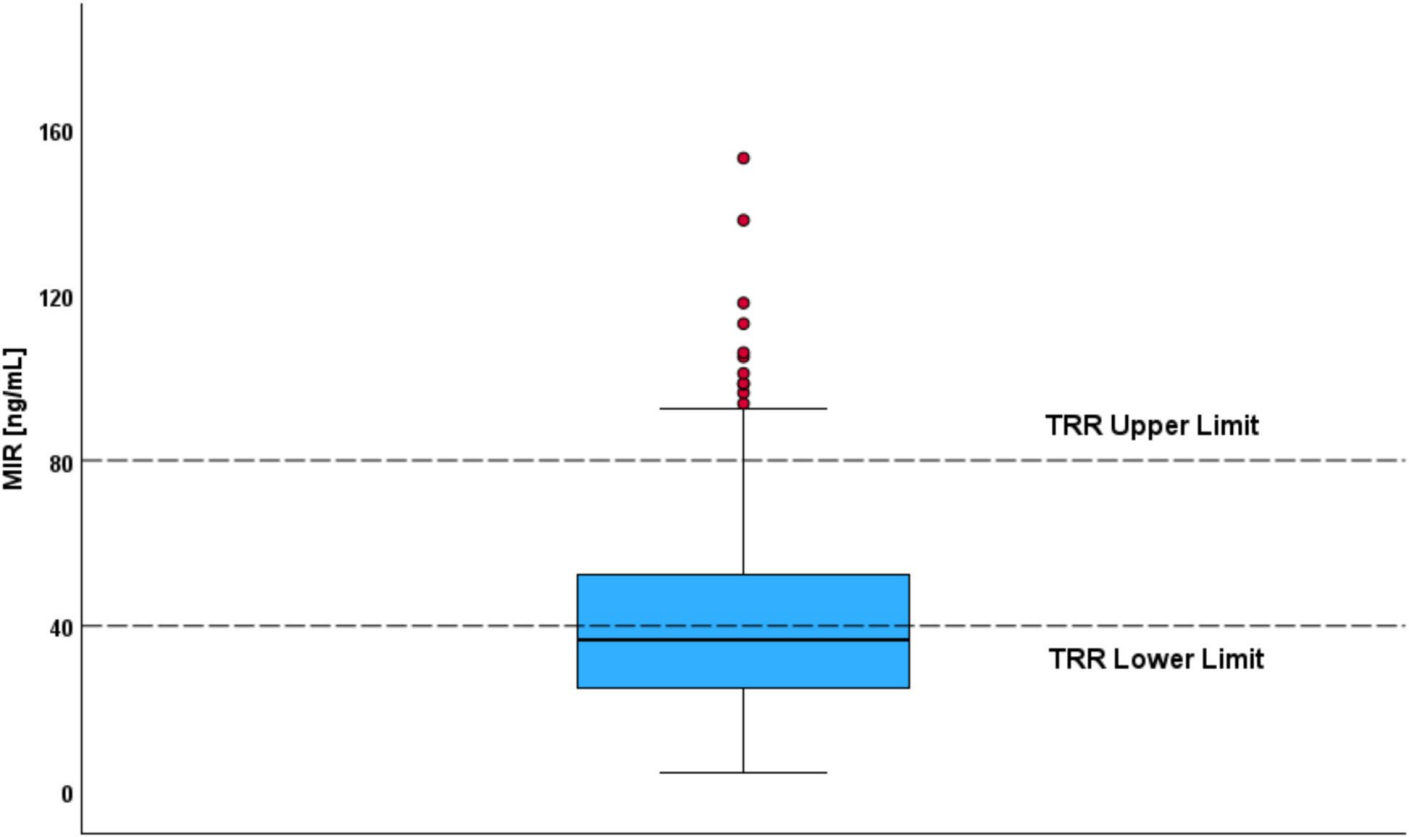
Therapeutic reference range (TRR) and distribution of mirtazapine (MIR) concentrations. Boxplot showing measured plasma concentrations of MIR. The TRR is indicated by horizontal lines at 30.0 ng/mL (lower limit) and 80.0 ng/mL (upper limit). The central line within the box represents the median concentration (36.8 ng/mL), while the box itself spans the interquartile range from the 25th percentile (25.0 ng/mL) to the 75th percentile (52.5 ng/mL). Red dots denote outliers, defined as values above the upper whisker, most of which exceed the therapeutic range

We compared our dataset with the indicated reference ranges from Hiemke et al. [19]. Based on the indicated TRR [30.0–80.0 ng/mL], our calculated median MIR concentration was 36.8 ng/mL. Only approximately half of the patients exhibited adequate concentrations of their medication with MIR. Around 38% of the samples had concentrations below the TRR (< 30.0 ng/mL), and 8% had concentrations exceeding the TRR area (> 80.0 ng/mL) (shown in Fig. 1). Patients below the TRR were significantly younger than those above it (median age 51 years, IQR: 41–62 versus 64 years, IQR: 54–78; *p* = 0.002). Serum concentrations of MIR were significantly lower in smokers compared to non-smokers (median 33.5 ng/mL, IQR: 22.9–47.2 versus 38.9 ng/mL, IQR: 26.7–60.2, *p* < 0.001). For the chi-square analysis, MIR doses of 15.0 mg, 22.5 mg, 30.0 mg, 37.5 mg, and 45.0 mg were included. A significant association was found between MIR dose and TRR classification, with lower doses more frequently linked to subtherapeutic levels and higher doses to therapeutic or supratherapeutic concentrations (chi^2^ = 80.9, *p* < 0.001). In total, 76 patients (23%) received low daily doses of 15.0 mg or 22.5 mg.

### (IIa) C/D ratio

Sex- and age-related differences in C/D ratios were observed in our cohort. The median C/D ratio for the entire population was 1.2 (ng/mL)/mg, with women exhibiting about 18% higher C/D ratios of MIR compared to men (1.1 (ng/mL)/mg, IQR: 0.8–1.4 in males versus 1.3 (ng/mL)/mg, IQR: 0.9–1.7 in females), indicating gender–related differences in MIR metabolism (*p* < 0.001).

Elderly patients, defined as aged above 65 years, demonstrated approximately 27% higher C/D ratios for MIR (1.4 (ng/mL)/mg, IQR: 0.8–1.9) if > 65 years versus 1.1 (ng/mL)/mg, IQR: 0.9–1.5 in < 65 years.A significant positive correlation was observed between age and C/D ratio (Spearman’s *ρ* = 0.196, *p* < 0.001).

Smoking was associated with significantly lower C/D ratios, with a median of 1.0 (ng/mL)/mg (IQR: 0.8–1.3) in smokers compared to 1.3 (ng/mL)/mg (IQR: 1.3–1.7) in non–smokers (*p* < 0.001).

### (IIb) DRC

**Fig. 2:**
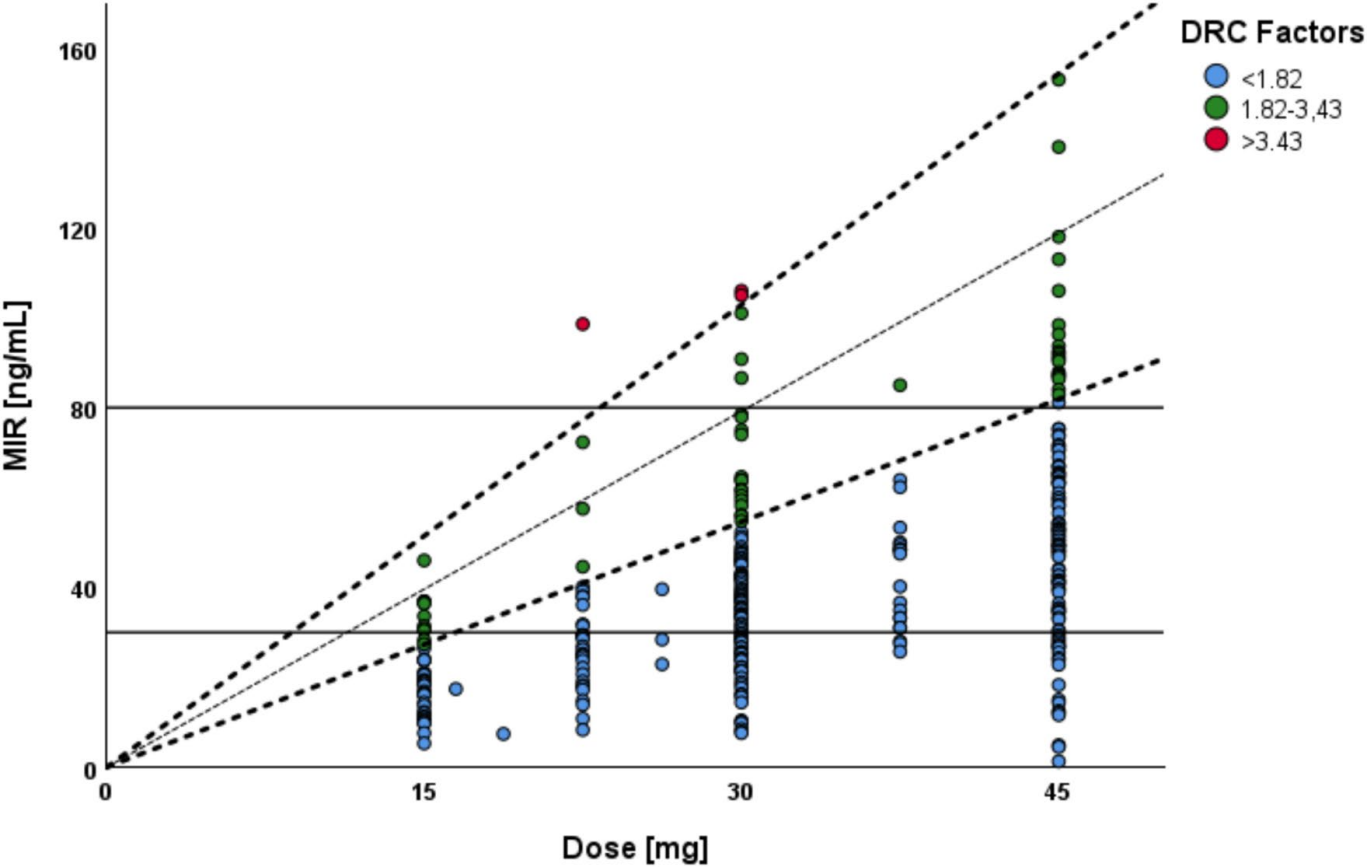
Dose–related concentration (DRC) of mirtazapine (MIR)

Our findings indicate a significant difference between the previously established DRC factors and those derived from our dataset. According to the reference range provided by Hiemke et al. (DRC mean = 2.63, DRC low = 1.82, DRC high = 3.43), only 15% of patients in our cohort had DRC values within the expected range (shown in Fig. 2). Additionally, when patients with known CYP3A4 or CYP2D6–interacting medications were excluded (*n* = 272), the distribution remained similar, with only 11% of patients falling within the reference range. Notably, 84% of patients had DRC values below 1.82, while only three patients (1%) had values exceeding 3.43. The mean DRC calculated for our cohort was 1.28. Administered MIR doses are plotted on the x-axis, the measured MIR serum concentration on the y-axis, and the DRC ranges (blue dots refer to < 1.82, green dots to 1.82–3.43, and red dots to > 3.43) are represented as the third axis. The illustration shows the distribution of the samples according to the DRC ranges defined by Hiemke et al. [19], categorized into low (< 1.82), mean (1.82–3.43), and high (> 3.43) DRC values.

### (III) MR

**Fig. 3:**
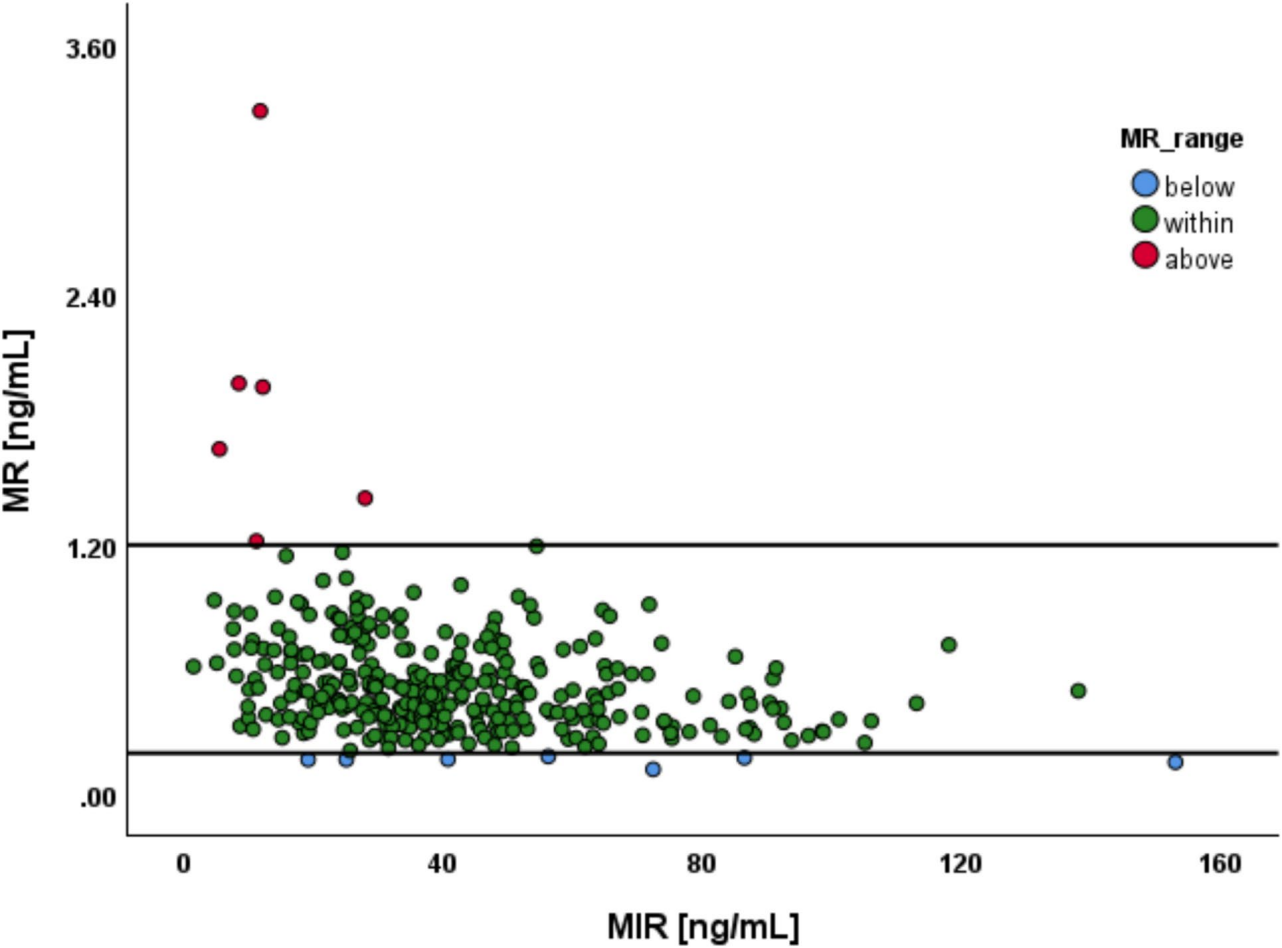
Metabolic ratio (MR) of mirtazapine (MIR). Distribution of samples categorized into three groups, based on the MR (0.2–1.2 ng/mL): below the range (blue dots), within the range (green dots), and above the range (red dots)

In total, 13 samples (4%) exceeded the previously suggested MR range of 0.2–1.2 ng/mL. Distribution is shown in Fig. 3.

The MR remained consistent across age groups, with median values of 0.5 ng/mL IQR: 0.4–0.6) for patients over 65 years and 0.5 ng/mL (IQR: 0.4–0.7) for those under 65 years (*ρ* =  − 0.056, *p* = 0.312). When comparing sex, women exhibited significantly higher MR values than men, with a median of 0.5 ng/mL (IQR: 0.4–0.7) compared to 0.4 ng/mL (IQR: 0.3–0.6) in men (*p* < 0.001), corresponding to a 25% difference. MR values remained consistent across smoking groups, with a median of 0.5 ng/mL (IQR: 0.4–0.7) in smokers and 0.5 ng/mL (IQR: 0.4–0.7) in non–smokers (*p* = 0.281).

## Discussion

In the present study, our aim was to investigate and confirm the TDM tools TRR, C/D ratio, DRC, and MR in patients treated with MIR for major depression in a real-world naturalistic setting. This structured TDM report may provide a clear framework for improving treatment decisions in consideration of dose adjustments, and consequently patient outcomes.

### (I) TRR – Observed distribution of MIR serum levels

The manufacturer’s recommended therapeutic dosage for MIR ranges from 15.0 to 45.0 mg daily. The previously recommended target range (40.0–80.0 ng/mL) [33] was modified in 2017 (30.0–80.0 ng/mL) [41]. In comparison, Myung [25] and Meineke et al. [36] reported respective median concentrations of 43.6 ng/mL and 37.0 ng/mL (15.0 mg of MIR), which are consistent with our findings (36.8 ng/mL). In contrast, Shams et al. [10] reported a notably lower median of 19.5 ng/mL, attributing this to factors such as older age and drug interactions. Establishing a minimum effective concentration threshold of 30.0 ng/mL facilitates the adjustment of MIR treatment doses, given that a significant portion of responders demonstrated serum concentrations exceeding this limit. In our patients, we observed that the distribution of dosing was closer to the mean value compared to the manufacturer’s recommended range of 15.0 to 45.0 mg daily, with a mean daily dose of 32.9 mg. This is particularly notable, as 38% exhibited serum concentrations below the recommended TRR of 30.0 ng/mL. Depressive patients undergoing MIR treatment may face a diminished response risk if their MIR plasma concentrations fall below 30.0 ng/mL [42]. Various factors within the natural setting of this study may have contributed to the variability in the range of serum concentrations of MIR, including sex, age, and concurrent medication [5]. Smoking is another relevant factor, as it was associated with significantly lower MIR serum concentrations in our cohort, likely due to CYP1A2 and CYP3A4 enzyme induction [43]. Furthermore, our data suggest that lower prescribed doses are linked to a higher proportion of subtherapeutic serum levels. This includes a considerable subgroup, 23% of our cohort, treated with low-end daily doses of 15.0–22.5 mg, which may partially explain the 38% of patients with MIR concentrations below the TRR [44]. Age may have influenced serum levels, as younger patients were more likely to fall below the TRR, consistent with a higher hepatic clearance in younger adults [44, 45].

#### (IIa) C/D ratio — impact of age, sex, and smoking status

Our findings for the median C/D ratio of 1.2 (ng/mL)/mg are consistent with previously reported values (1.5 (ng/mL)/mg [25]), while other studies have reported C/D ratios higher in females (20%) and higher in elderly patients (24% in > 60 years) [46]. In our cohort, women exhibited significantly higher C/D ratios compared to men, which is likely due to sex-related differences in hepatic clearance and MIR metabolism [44, 47]. Elderly patients over 65 years of age exhibited an increased C/D ratio of MIR, which is likely due to polypharmacy and a natural age-related decline in hepatic drug clearance, which can lead to reduced metabolic capacity and prolonged drug elimination [10, 26, 36, 48]. This observation is supported by the significant positive correlation between age and C/D ratio observed in our cohort. As previously described by Myung and Tveit et al. [25, 27], we can therefore confirm sex- and age-related differences. However, there were no variations in administered drug doses between elderly and younger patients and between women and men. This supports the general observation that older individuals, particularly those over 65 years, may require lower doses of antidepressants to achieve therapeutic effects similar to those seen in younger adults.

In addition, smoking status emerged as a relevant factor. Smokers exhibited significantly lower C/D ratios compared to non–smokers, suggesting enhanced MIR clearance in this group. This finding aligns with the known induction of hepatic enzymes, particularly CYP1A2 and CYP3A4, by components of cigarette smoke, which may accelerate MIR metabolism and thereby lower serum concentrations. This observation is consistent with prior findings [37, 49, 50], which both demonstrated significantly reduced MIR serum concentrations and C/D ratios in smokers, attributing the effect to smoking-induced hepatic enzyme activity.

#### (IIb) DRC — variations between guidelines andreal-world data

This study assessed the feasibility of the previously published DRC factors, which were calculated based on pharmacokinetic parameters, including bioavailability and clearance [19]. Differences in DRC values from the guideline-recommended ranges were observed. Since the previously established DRC factors were calculated based on bioavailability, total body clearance, and elimination rate constant, they only aligned with a limited portion of our cohort. Our findings challenge the guideline assumption that approximately two-thirds of patients fall within the normal DRC range. In our cohort, only 15% of patients had DRC values within the expected range (DRC low = 1.82, high = 3.43), indicating a significantly higher proportion of deviations from previously reported data. Variations may stem from differences in real-world patient populations, such as a higher mean daily dosing (32.8 mg) but unexpectedly low serum concentrations in 38% of patients. Possible explanations include altered drug metabolism, polypharmacy, an extended time interval between medication intake and blood sampling, or reduced bioavailability in certain subgroups. To exclude the influence of polypharmacy, we conducted a subgroup analysis. Patients receiving medications with CYP3A4 (e.g. carbamazepine, ciprofloxacin) or CYP2D6 (e.g. fluoxetine, bupropion, haloperidol) interactions were removed from the cohort. The distribution of DRC values remained similar, with only 11% of patients falling within the expected range. This suggests that polypharmacy alone does not fully explain the observed deviations. Importantly, our analysis did not account for pharmacokinetic parameters, which may also contribute to the observed discrepancies. Further studies in an independent naturalistic cohort are needed to determine the clinical relevance of these findings.

#### (III) MR – identifying metabolic outliers

In our study, we observed that women had a higher ratio of NDM to MIR compared to men. This may be explained by sex-related differences in hepatic metabolism, as CYP3A4 is the primary enzyme responsible for the demethylation of MIR to NDM [51], and women generally exhibit higher CYP3A4 activity than men [52]. The MR, calculated as the NDM/MIR concentration ratio, exclusively reflects CYP3A4 activity. MIR is also metabolized by CYP2D6 and CYP1A2, whose activity may vary between individuals and contribute to interindividual variability [53, 54]. However, these enzyme activities are not captured by our MR analysis, as it exclusively reflects CYP3A4-mediated demethylation. This observation is consistent with previous reports indicating that patients with NDM/MIR ratios below 0.4 experienced significantly more side effects compared to those with higher ratios [10]. We found that the median MR remained consistent across age groups, with no statistically significant difference observed between elderly individuals (> 65 years) and younger patients. This suggests that age may not substantially influence the MR of MIR in clinical practice. While some pharmacokinetic studies describe reduced hepatic clearance and altered exposure levels of MIR in elderly individuals, our findings in this cohort do not support an age-related effect on MR [44]. No significant difference in MR was observed between smokers and non-smokers. This is due to the definition of MR, which reflects NDM, the primary CYP3A4-mediated metabolite of MIR. In contrast, 8–OH–MIR, formed mainly by CYP1A2 and influenced by smoking, takes no part in our MR. In contrast to the findings reported by Lind et al. [37], we did not observe a significant difference in MR between smokers and non-smokers. In summary, unlike the C/D ratio, the MR shows no influence of smoking, as it exclusively reflects CYP3A4 activity. It therefore serves as a complementary measure to C/D, and together they provide a more comprehensive view of CYP enzyme activity.

There are some limitations of this study that should be considered. First, a major limitation is the lack of clinical outcome data, which prevents establishing a direct correlation between serum MIR concentrations and treatment efficacy or adverse effects, and makes it difficult to assess whether TDM-based dose adjustments improve patient outcomes [55]. Second, CYP genotyping was not performed; however, it is not recommended according to pharmacogenomics sources CPIC and PharmGKB [56]. Furthermore, Tveit et al. [27] found no clinically meaningful association between CYP2D6 genotype and dose-adjusted serum concentrations (C/D ratios) of MIR, reinforcing that genotyping does not contribute to dose optimization. Therefore, routine CYP genotyping shows no benefit in clinical utility guiding MIR therapy. Third, despite supervised drug administration ensuring adherence, interindividual variability in drug metabolism and polypharmacy effects remain potential influencing factors.

Therefore, this study remains a guideline-compliant and realistic setting for evaluating TDM data for MIR without the need for CYP genotyping. Currently, no dose reduction for MIR is recommended in older individuals, and its effect on QT prolongation has been found to be negligible [57].

The observed increase in the C/D ratio with age may be related to reduced hepatic clearance. Although polypharmacy was considered in the analysis, the absence of detailed information on all concomitant medications limited our ability to fully assess its specific impact on MIR pharmacokinetics.

A notable strength of this study is its substantial sample size, enabling a comprehensive analysis of C/D ratio differences between older and younger individuals, as well as identifying the onset of these changes and their progression with advancing age. Interestingly, the proportion of female patients in our inpatient cohort was relatively low (42%), which differs from prior studies where a higher proportion of female patients is often observed. Additionally, the availability of data on the metabolite NDM provided further depth to our analysis, enhancing our ability to interpret age-related pharmacokinetic differences. As drug administration took place under supervision in an inpatient setting, patient adherence could be reliably ensured. This controlled environment minimizes the risk of missed doses or intentional non-adherence, thereby strengthening the reliability of serum concentration measurements. Notably, TDM revealed one likely non-adherent patient, even though drug administration was supervised. Extremely low serum concentrations were observed below the lower limit of quantification. This individual case would have remained undetected without TDM. However, collecting data on the primary MIR metabolite NDM may serve as an adherence indicator, as NDM, unlike MIR, cannot be conveniently ingested just before the measurement due to its metabolization process. As a final consideration, to avoid inaccurately low drug concentrations caused by adsorption to polymer gel in separator tubes, all samples were collected using plain serum tubes without gel separator. This preanalytical known issue affects lipophilic compounds such as MIR and was thereby effectively avoided, ensuring the reliability of serum concentration measurements [58].

The outcomes presented in this study expand our understanding of MIR levels in a near–patient setting and thus provide clinicians with insights to improve the long-term treatment of patients with major depressive disorder. The integration of TDM findings into clinical practice facilitates the determination of optimal MIR dosing and its adjustment for specific groups, including dependence on age and sex.

In order to interpret outliers potentially caused by an unusual daily dose, abnormal MR, or partial non-adherence, it is beneficial to use TDM tools such as C/D ratio and MR. However, the routine application of TDM for MIR might be uncertain, given the lack of a positive correlation between serum concentration and clinical response, as indicated by Hiemke [19]. Conducting pharmacokinetic studies by linking different TDM tools becomes crucial when aiming to comprehend inter- and intraindividual variations in pharmacokinetics among real-world patients undergoing prolonged treatment. Age-related physiological changes, including reduced hepatic metabolism and renal clearance, can lead to altered drug exposure in elderly patients. Adjusting the C/D ratio for age allows for a more individualized dosing approach, potentially reducing the risk of subtherapeutic or toxic concentrations. These insights lay the foundation for evaluating the broader clinical utility of TDM in MIR therapy.

## Conclusion

This study provides a well-founded dataset, demonstrating the applicability of therapeutic TDM tools for MIR in routine clinical practice. Observed age- and sex-related pharmacokinetic differences emphasize the importance of individualized dosing strategies. Moreover, we observed notable deviations in DRC from established guideline expectations. While these findings offer valuable real-world insights, further multicenter studies are needed to confirm their reproducibility and assess their broader clinical relevance.

## Data Availability

The datasets generated and analyzed during the current study are not publicly available due to ethical restrictions, as they contain sensitive patient information. However, they are available from the corresponding author in anonymized form on reasonable request and with permission from the Ethics Committee of the University Hospital LMU Munich.
